# Retinoblastoma and vision

**DOI:** 10.1038/s41433-021-01845-y

**Published:** 2022-01-05

**Authors:** Omar Warda, Zishan Naeem, Kelsey A. Roelofs, Mandeep S. Sagoo, M. Ashwin Reddy

**Affiliations:** 1 Retinoblastoma Service, Royal London Hospital, London, UK; 2 Ocular Oncology Service, Moorfields Eye Hospital, London, UK; 3 NIHR Biomedical Research Centre for Ophthalmology at Moorfields Eye Hospital and University College London Institute of Ophthalmology, London, UK; 4 Queen Mary University of London, London, UK

**Keywords:** Eye cancer, Outcomes research

## Abstract

The assessment of vision has a growing importance in the management of retinoblastoma in the era of globe-conserving therapy, both prior to and after treatment. As survival rates approach 98–99% and globe salvage rates reach ever-higher levels, it is important to provide families with information regarding the visual outcomes of different treatments. We present an overview of the role of vision in determining the treatment given and the impact of complications of treatment. We also discuss screening and treatment strategies that can be used to maximise vision.

## Abstract (zh)

在全球保守治疗视网膜母细胞瘤的时代中, 治疗前后的视力评估在视网膜母细胞瘤的管理中日益重要。随着生存率接近98-99%, 全球挽救率达更高水平, 向家庭提供不同治疗方法的视力结局的信息非常重要。我们概述了视力在决定治疗方式和治疗并发症的影响中的作用。我们还讨论了筛查和治疗策略, 以最大限度地提高视力。

## Introduction

The management of retinoblastoma (RB) is guided by a set of hierarchical goals, of which the most important is saving the child’s life. In the nineteenth century, enucleation was the only method of treating retinoblastoma [1], and to this day it remains the treatment of choice in some instances, particularly in children presenting with neovascular glaucoma or those with large tumours [2].

In the 1920s external beam radiotherapy (EBRT) was introduced in an attempt to salvage the globe, and it was widely used until the early 2000s [3]. Although the recognition of an increased risk of subsequent primary neoplasms (SPN) ultimately led to its limited use [3, 4], during those years the armamentarium of alternative globe-conserving treatments expanded tremendously. An improvement in 5-year survival from 85% in the early 1960s to upwards of 98% today has led to a greater focus on reducing the morbidity associated with treatment, firstly by increasing the rate of globe salvage, and secondly by aiming to optimise visual outcomes.

As part of a holistic approach, it is essential for clinicians to understand the impact of various treatment modalities on vision, as severe visual impairment, particularly in bilateral cases, can negatively impact the child’s development and overall well-being [5]. Although family counselling often appropriately centres on saving the child’s life, it is important not to let the consideration of visual outcome fall by the wayside, as this is an additional piece of information that families find useful to help guide their decisions [6, 7].

The purpose of this review is to discuss the implications of various treatment modalities used in RB on vision, to review the management of amblyopia in children with RB and to discuss potential avenues for optimisation of visual outcomes in the future.

## Assessing vision in infants/children

Visual assessment in infants can be difficult. While Snellen visual acuity is used by many studies in the ophthalmic literature, this method of quantification requires children who are old enough (often over 5 years) to co-operate. Given that RB primarily affects infants and young toddlers, methods of vision assessment need to be tailored to this age group.

### Age-appropriate visual acuity (VA) assessment

Age-appropriate VA assessment can be performed using standard orthoptic techniques including Cardiff Cards, Keeler Cards, Kays picture tests and Single Sheridan Gardner Tests [8]. When possible VA should be assessed monocularly. An encouraging and animated approach is used to maximise the engagement and co-operation of the child during VA assessment. As children with RB grow older, the choice of VA assessment tool should similarly evolve to ensure the most refined quantification possible.

### Timing of VA assessment

At presentation, children with RB are often seen on an urgent basis at the retinoblastoma unit, which in many cases requires them to travel a significant distance. In our service, the visual assessment is performed by the orthoptist on presentation as well as at every ophthalmological evaluation during treatment; unless systemic chemotherapy, in which case visual assessment is performed after the final cycle. When RB is in remission, a visual assessment is performed at each follow-up appointment, this takes place before the examination under anaesthesia to avoid the child and family travelling great distances twice. The assessment can prove difficult particularly for starved children before their anaesthetics, so it may be necessary to settle for binocular VA in this instance. If the quantitative assessment is not possible, qualitative methods are used i.e. fixing and following a light and different-sized target to assess for a fixation preference.

We assess vision in a similar manner on subsequent follow-up visits. During the active treatment phase, the timing of visits depends largely on the treatment modalities employed. In the later years when there is no longer ongoing active RB treatment, the frequency of visits may in some cases be driven by the need to monitor for or manage amblyopia.

### Electrodiagnostic methods of assessing visual function

In addition to the subjective methods of assessing VA mentioned above, electrodiagnostic studies may occasionally be helpful. Electroretinograms (ERGs), visual evoked potentials (VEPs) and fundus fluorescein angiography (FFA) have been found to be particularly useful in assessing for retinal toxicity and visual loss following the use of intra-arterial chemotherapy in eyes with tumour-free foveolae [9, 10]. Whilst ERGs are recognised not to correlate with vision, VEPs have a role in infants where the foveola is not involved [9–11]. In fact, VEP Spatial Frequency is better than behavioural methods up to the age of 3 (11) and is a useful adjunct to behavioural methods in this age group when assessing novel treatments and their impact on vision.

## Early screening for at-risk infants

### Childhood screening for retinoblastoma

In addition to appropriate timely VA assessment, screening at-risk children plays an important role in optimising visual outcomes as early diagnosis correlates with smaller tumours [12]. Consensus-based guidelines for screening are stratified by risk category, with high-risk children being screened at 2 weeks of age and repeated every 2–4 weeks initially and then at progressively long intervals until 5–7 years of age. While low-risk screeners are examined primarily within 4 weeks and follow-ups are dependent on genetic testing and are often awake examinations [13].

### Prenatal screening and early-term delivery

With advances in genetic analysis, some groups have investigated the role of prenatal diagnosis via amniocentesis. In a retrospective study, Soliman et al found that compared to traditional postnatal screening, infants that underwent prenatal diagnosis with early-term delivery (36–38 weeks) had better vision outcomes and required less invasive therapy [14].

### The role of screening for earlier presentation in babies with RB1 pathogenic variants

Risk assessment and stratification involving genetics and counselling is the basis for screening children at higher risk of development of retinoblastoma [12]. These babies have examinations under anaesthesia from birth at regular intervals. Early diagnosis results in the detection of smaller tumours and this maximises survival, visual outcomes and reduces the need for chemotherapy, radiotherapy and enucleation [11]. Imhof et al reported that in their screening programme in the Netherlands, 90% of patients had final visual acuity of 6/6 to 6/12, because binocular macular involvement was rare [15].

A consensus statement from the American Association of Ophthalmic Oncologists and Pathologists in 2017 agreed on the frequency of thorough dilated fundus examination of children stratified according to high, intermediate and low-risk screeners [16].

## The impact of unilateral enucleation on visual function and child development

Enucleation can be required either at presentation (primary) or following the failure of other therapies (secondary enucleation). In our practice, we typically recommend primary enucleation for patients with large tumours and buphthalmos/neovascular glaucoma (Group E or cT3c on AJCC classification) [17]. This recommendation is based both on the fact that these eyes often have a very poor visual prognosis and, more importantly, that 50% will have evidence of adverse histopathology following enucleation [18]. Thus, removal of eyes falling into this category allows identification of which patients would benefit from adjuvant systemic chemotherapy and likely does not alter the child’s binocular visual functioning in a meaningful way. We do not routinely use neoadjuvant chemotherapy in buphthalmos, however, if there is an advanced orbital disease or gross optic nerve involvement suggested on MRI imaging, it will be considered. This is rare in the UK and it is seen more often in low-income countries with late presentation. Of course, there are instances when eye salvage is considered for children without buphthalmos and neovascular glaucoma, and in these cases, a discussion about visual potential is an important aspect of family counselling. Over the past two decades, reliance on enucleations has decreased at our institution. In the 1990s 80% of retinoblastoma patients underwent primary enucleation [19]. This decreased to 60% in the early part of the last decade [20], and now represents less than 40% of retinoblastoma cases.

It is important for clinicians to be aware of the implications of enucleation both from a psychosocial perspective and also with respect to the impact of monocular vision after unilateral enucleation. As this topic has become more relevant over the past decade paralleling the advances in globe salvage therapies, knowledge of the motor function of survivors of retinoblastoma is somewhat limited. Some series have found similar motor skills between children with RB aged six months to five years and controls [21]. On the other hand, others have shown that children with monocular vision due to retinoblastoma have difficulties with motion processing and motor skills and noted that approximately 40% of children aged three years and younger, who had one eye enucleated, were referred to early intervention clinics for visual‐motor‐coordination deficits [22]. As one may expect, similar delays in motor functioning have also been described in children with unilateral congenital cataracts [23].

However, it is equally important to consider the negative impact that globe salvage can have on a child’s development. Our group has shown that patients with group D eyes [24] undergoing enucleation have 1/3 fewer examinations under anaesthesia (EUAs) compared to those managed conservatively. This is particularly relevant given the potential toxicity of anaesthesia to the developing brain, as induction of apoptosis and interference with neural genesis have been demonstrated in animal studies. Moreover, the neurotoxicity of anaesthetics appears to be greatest during the period of maximal synaptogenesis, which generally occurs between 34-weeks gestation and 24 months of age [25].

## Globe-conserving treatments and their associated visual outcomes

### Systemic chemotherapy

**Fig. 1:**
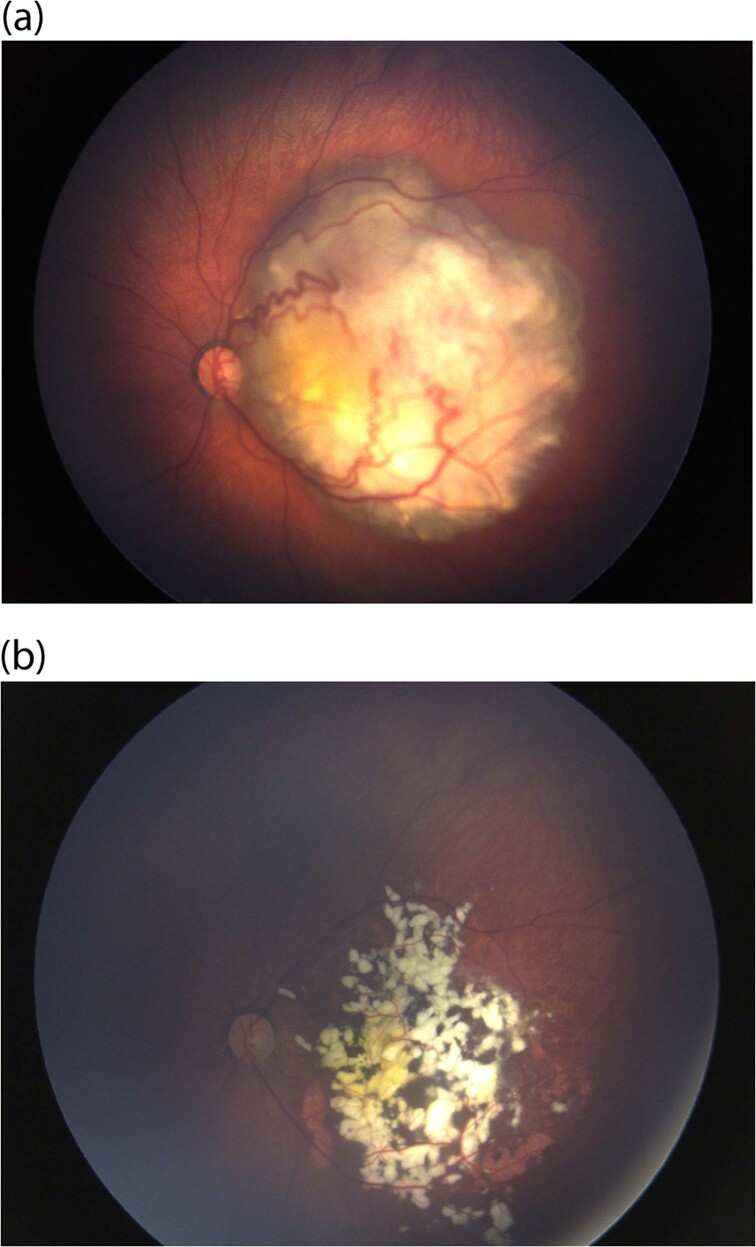
The role of systemic chemotherapy as primary treatment. **a** Retinoblastoma group C in the left eye at presentation. **b** Good response following systemic chemotherapy with calcified-treated tumour (Type 1 regression).

Intravenous chemotherapy (IVC) was introduced in the mid-1990s [26] at our centre due to pioneering work by Dr Judith Kingston and quickly revolutionised the management of retinoblastoma. Use of IVC results in globe salvage rates of 90% of group A–C eyes [27] and recently 63% of group D eyes [28]. Thus, attention towards minimising visual morbidity has increased [29]. Systemic chemotherapy can result in retinal detachments and haemorrhages in large tumours which could have a negative effect on potential vision. However, a reduction in vision from presentation rarely occurs, as retinal detachments at presentation typically resolve with systemic chemotherapy as the tumour responds [25, 26]. Figure 1 demonstrates the role of this treatment on a tumour and its role in salvage.

#### Tumour location as a predictor of visual outcome

Although VA > 6/60 is achieved in the majority of cases treated with IVC (71%), the visual outcome can be better predicted by taking tumour features into account. Desjardins et al. and Kim et al. both reported that tumour location was the strongest prognostic factor of vision [15, 30]. As would be expected, foveal involvement and a greater number of tumours is associated with poorer visual outcomes [31]. All eyes with maculopathy caused by RB had a post-treatment acuity of 6/60 or less. Other studies concluded that all tumours outside the fovea had an acuity of 6/12 or better, whereas all tumours in the fovea had an acuity of 6/18 or worse [32]. Despite this, macular location does not always portend a dismal visual prognosis, with some series reporting that 33% of treated tumours within 1.5 DD of the fovea achieving a VA of 6/12 or better [33].

#### The impact of tumour regression patterns on visual outcome

Regression patterns were initially reported in RB patients who were treated with EBRT [34]. Regression patterns have changed as chemotherapy has replaced EBRT as the treatment of choice for retinoblastoma but the terminology is still commonly used. These patterns are summarised in Table 1 [35].

**Table 1:** Regression patterns observed in retinoblastoma.

TYPE	Regression pattern
Type 0	No visible remnant
Type 1	Completely calcified remnant
Type 2	Completely noncalcified remnant
Type 3	Partially calcified remnant
Type 4	Atrophic chorio-retinal flat scar

Familiarity with tumour regression patterns is important in differentiating tumour regression from incomplete response or tumour recurrence. With regression, RB becomes smaller in size with stable margins and typically attains some degree of calcification. Judgement of regression is challenging, as some tumours become completely calcified, whereas others have minimal or no calcification.

Studies reporting the distribution of regression patterns of retinoblastoma treated with chemotherapy was 2–3% for type 0 regression, 10–13% for type I, 3–5% for type II, 23–33% for type III, and 51–57% for type IV [18, 36]. It has been reported that eyes with type IV regression patterns tended to have better final visual outcomes, which was associated with size and location of the tumour [15, 16].

#### Tumour staging and visual outcome

The classification and staging of the tumour at presentation was reported to be one of the prognostic factors affecting long-term visual outcomes after systemic chemotherapy. The International Intraocular Retinoblastoma Classification (IIRC) (as shown in Table 2) has been developed through international collaboration to stage intraocular disease with respect to the prognosis of primary chemotherapy and focal therapy [37]. Long-term control was good for groups A/B/C tumours, but not for groups D/E tumours due to better response to chemotherapy in those groups, thus, affecting visual outcomes [18, 23, 38]. Group E eyes were enucleated at presentation.

**Table 2:** IIRC classification system for intraocular retinoblastoma [98].

	International Intraocular Retinoblastoma Classification (IIRC)
Group A (very low risk)	All tumours are 3 mm or smaller, confined to retina and at least 3 mm from fovea and 1.5 mm from optic nerve. No seeding allowed
Group B (low risk)	No seeding and discrete retinal tumour of any size and location. A small cuff of subretinal fluid extending ≤5 mm from the base of the tumour
Group C (moderate risk)	Focal vitreous or subretinal seeding and discrete retinal tumours of any size and location. Any seeding must be local. Up to one quadrant of subretinal fluid may be present
Group D (high risk)	Eyes with diffuse seeding and/or massive, non-discrete endophytic or exophytic disease. >1 quadrant of retinal detachment
Group E (very high risk)	Eyes that have been destroyed anatomically or functionally with one or more of the following: Irreversible neovascular glaucoma, massive intraocular haemorrhage, aseptic orbital cellulitis, tumour anterior to anterior vitreous face, tumour touching the lens, diffuse infiltrating retinoblastoma and phthisis or pre-phthisis

Bartuma et al. in 2014 studied 46 eyes, 7 group A, 23 group B, 3 group C, 11 group D and 2 group E, and concluded that 73% had expected visual acuity, using size of the tumour, macular involvement and adjuvant treatment to the retina [33].

#### Age at presentation and visual outcome

Interestingly, the age of the child had a significant association with the type of regression pattern seen after treatment. Type IV pattern was associated with younger age at presentation, whereas other regression patterns were noted in older children which has a direct effect on the visual outcome as type IV regression is associated with better visual prognosis [39]. This could be related to the size and early detection of tumours, with smaller tumours treated with focal treatment in an anterior location rather than IVC.

### Intra-arterial chemotherapy

Recent years have seen continued innovation in search of a globe salvage treatment that could retain vision without the risk of metastasis. The past decade has seen a shift in the management of RB with the introduction of intra-arterial chemotherapy (IAC), a local, high dose chemotherapy regimen delivered directly via the ophthalmic artery. Many units around the world use IAC for RB [40]; however, the lack of safety profile data has delayed its unanimous acceptance [41–44]. It is usually given initially following presentation (primary) or following systemic chemotherapy when the tumour(s) are refractory to treatment (secondary).

**Fig. 2:**
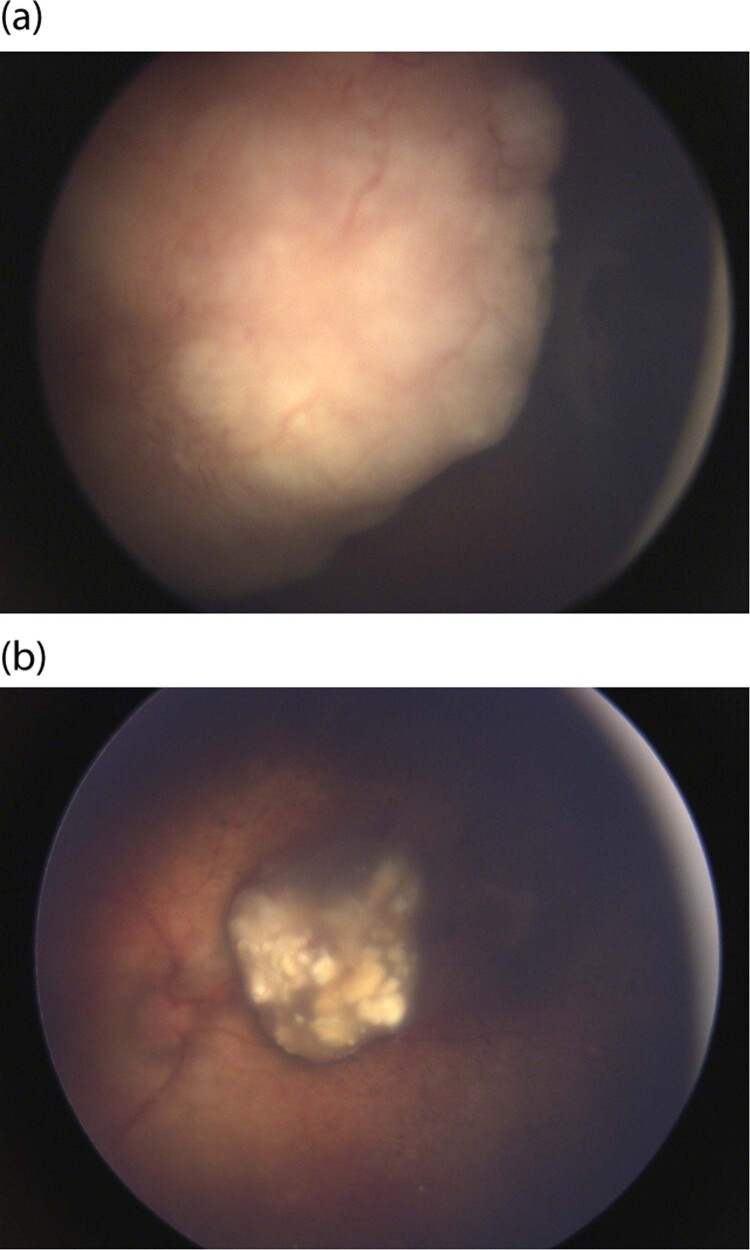
The role of intra-arterial chemotherapy as primary treatment. **a** Nasal retinoblastoma tumour in a group D eye at presentation with obscuration of the optic nerve. **b** Tumour treated with primary intra-arterial chemotherapy showing shrinkage of the tumour (Type III regression) with sparing of macula and good visual outcome.

Intra-arterial chemotherapy was first used in Japan [45], and later more widely popularised by David Abramson in New York [46]. Initial reports did not document any cases of iatrogenic visual loss [47] and Abramson and colleagues went on to report a ‘super-selective’ method of delivering melphalan directly to the ophthalmic artery [42]. While several studies have confirmed high tumour control rates (Fig. 2) [26, 27, 47, 48], more widespread usage has identified iatrogenic side effects, including chorio-retinal ischaemia and atrophy, questioning the value of salvaging eyes if treatment was associated with a guarded visual potential [25, 49, 50]. This was highlighted by Yousef et al. [41] in their systematic review of Intra-Arterial Chemotherapy [12].

**Fig. 3:**
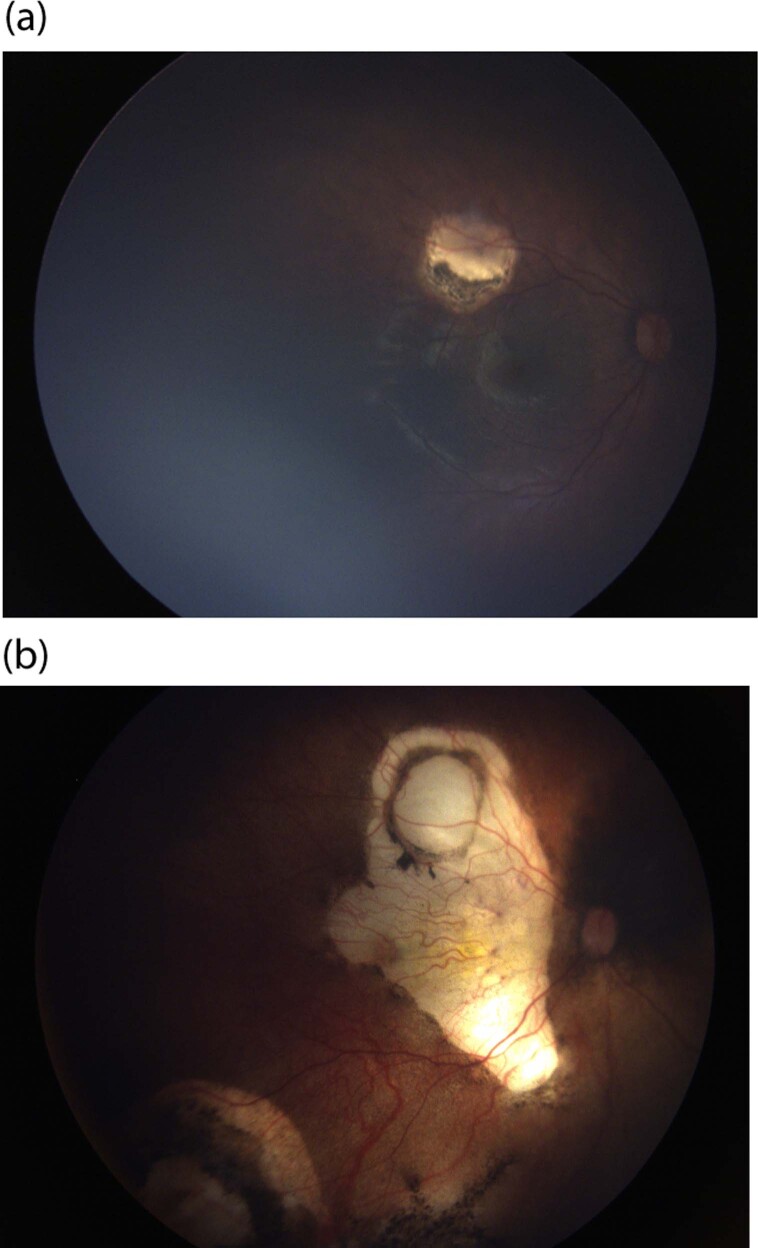
The impact of choroidal ischaemia from intra-arterial chemotherapy on vision. **a** Healthy fovea before IAC for several relapsing peripheral lesions uncontrolled by focal therapy. **b** Choroidal ischaemia after IAC with visual loss.

The main reason for vision loss following IAC is the development of choroidal ischaemia in the previously unaffected foveolar area (Fig. 3). The exact cause is somewhat unknown, but melphalan toxicity and/or difficulties in catheterisation of the ophthalmic artery [51] may be responsible.

The predisposition to ocular toxicity after IAC still avoids enucleation of the eye and many parents would like to avoid enucleation even if ocular toxicity and visual loss was a risk [52]. The difficulties associated with the monocular vision from eye removal are highlighted above and there are potential psychosocial consequences that distress families. Using the super-selective technique for refractory tumours where the catheter is placed into the ophthalmic artery, there was at 42% risk of visual loss. In order to ascertain this figure, only patients with healthy foveolae were included so all patients should have the potential for good vision. Despite the small number (12 patients), visual loss could be attributed to the procedure. Visual loss was quantified using VEPs in order to provide objective measures of vision in the cohort (median age at first IAC was 28 months). Three factors were considered important determinants of the risk of visual loss: prior radiotherapy, dose of melphalan and catheterisation complications [34].

#### Dose adjustment of intra-arterial melphalan

Melphalan dosage appears to be a critical factor in visual loss following IAC, more so than complications from the catheterisation itself. In light of this, our group investigated the utility of age-adjusted melphalan dosing and found that this protocol reduces the risk of visual and ocular motility complications while maintaining excellent salvage rates for secondary IAC [53]. Reddy et al demonstrated that there was no visual loss in the cohort of 9 patients with previously healthy foveolae with such a strategy [49]. This was subsequently proven in animal models and patients by Daniels et al. using doses of 0.4 mg/kg [54]. It is still uncertain as to whether a weight-appropriate dose or an age-appropriate dose is the best approach to treat tumours and avoid complications [41, 42].

### Intravitreal chemotherapy

Intravitreal injection of chemotherapy (IViT) was originally used by Ericsen and Rosengren in 1960 using thiotepa with indefinite conclusions. Melphalan was used in Japan but there were concerns regarding the safety of this approach [55]. Several reports have suggested that the risk of tumour dissemination is very small, which can be reduced even more by using safety-enhanced injection techniques [56, 57]. However, other complications including retinal detachment, retinal and choroidal toxicity can result in permanent vision loss and precipitate enucleation [58]. These have been classified by Francis Munier [59] with grades IV & V likely to reduce vision. Although globe salvage rates are increasing with the advent of intravitreal chemotherapy, visual acuity outcomes have not been extensively examined. Most series published to date focus on disease control and globe salvage; therefore, the impact of intravitreal therapy on visual function needs further attention [60].

### Laser

**Fig. 4:**
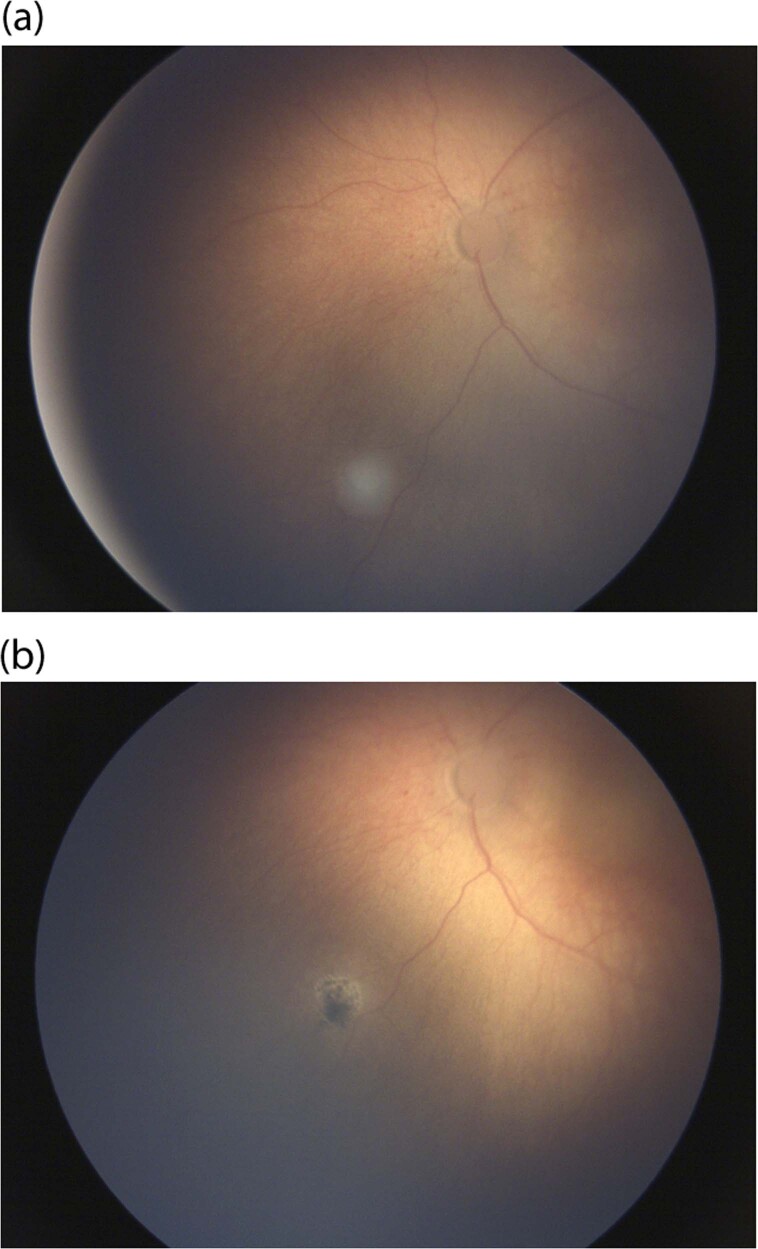
Laser treatment on a small tumour. **a** Small group A tumour at presentation in the left eye. **b** Group A tumour in the left eye treated with laser with good response.

A recent Cochrane review concluded that there are varying methods (focal or transpupillary thermotherapy), delivery systems (Argon 532 nm or Diode 810 nm) and settings (power and duration) used in treating RB, suggesting the delivery is dependent on the ocular oncologists’ preferences, rather than clinical trials [61]. Laser treatment is effective and peripheral lesions will not cause visual loss (Fig. 4).

Laser treatment to perifoveal retinoblastoma can reduce vision by either direct destruction of the fovea or secondary laser scar migration [38]. As a result, some centres avoid treating macular tumours with laser following chemotherapy [61] and use an extra-macular approach if the laser is to be used [62, 63]. As transpupillary thermotherapy (TTT) entails a long duration of energy delivery, some studies have found the use of TTT to be a poor prognostic factor for VA outcome, especially in macular tumours of IIRC group B eyes and D eyes [64–66].

The use of OCT to accurately locate the fovea and provide topographic macular assessment has allowed further refinement of focal laser therapy consolidation following chemotherapy [67–70]. Improved visual outcomes due to less foveolar damage and less scar migration have been demonstrated following OCT guided laser photocoagulation [42]. In this technique, a crescent-shaped boundary on the outer side of the tumour using a 532 nm laser can be performed sparing the foveal edge while still damaging the tumour blood supply to prompt regression, assuming that the foveal avascular zone will not contribute blood supply to the tumour. In some institutions, the choice of laser depends on tumour thickness; if <1 mm height, 532 nm laser is used and if >1 mm height 810 nm laser is preferred [71]. A recent study has shown that the results are broadly the same for chemotherapy with laser vs chemotherapy alone with regards to vision and macular tumour relapse [72]. Surprisingly, when systemic chemotherapy alone is directly compared with systemic chemotherapy and laser with either an extra-foveal [72] or extra-macular [63] approach, there was no statistical evidence of an increased relapse rate. Only one study [72] has directly compared vision with no laser (71 patients) vs foveal sparing laser (20 patients) and interestingly, there was no statistical difference in vision.

### Cryotherapy

Cryotherapy is often the focal treatment of choice for small peripheral RB tumours [73, 74]. Tumours less than 1.5 mm in diameter and 1.0 mm in thickness can generally be eradicated by one application of triple freeze-thaw cryotherapy. Tumours that are about 3.5 mm in diameter, 2.0 mm in thickness or both often require ≥1 session of cryotherapy.

As the use of cryotherapy is often away from the macula, one would expect good visual acuities for patients with healthy maculae. On the whole, the majority have visual acuities better than 6/12 [75]. However, as studies often combine cryotherapy with other treatment modalities conclusions regarding the specific impact of cryotherapy on VA can be difficult to delineate [47]. Side effects of cryotherapy are uncommon, but include vitreous haemorrhage, subretinal fluid, scarring and the treatment rarely can lead to rhegmatogenous retinal detachment [56].

### Radiotherapy

For many years external beam radiotherapy (EBRT) was the treatment of choice for RB. In bilateral disease, the more advanced eye was usually enucleated and the better eye was conserved with EBRT. Those patients suffered from long-term ocular co-morbidities, such as dry eye, contracted sockets with fat atrophy, cataract (if the lens could not be spared in the radiation field) and radiation retinopathy. Visual outcomes were poor [72]. Greater knowledge of these side effects and the attendant risk of subsequent tumour development after EBRT, such as sarcoma, encouraged the switch to primary systemic chemotherapy plus focal therapy for the past 2 decades [76–79].

Brachytherapy (plaque radiotherapy) is a method of delivering focal irradiation in order to minimise side effects to the surrounding tissues. The advantage is that the radiation dose is limited to the immediate vicinity of the tumour and its surroundings, thereby negating the risk of radiation complications such as further late-onset cancers. With regard to the eye, brachytherapy is most often used for intraocular malignancies such as uveal melanoma and retinoblastoma. Presently, Iodine^125^ and Ruthenium^106^ are the most commonly used radioisotopes for retinoblastoma [80, 81].

A study published by Carol Shields et al. in 2001 concluded that visually important radiation complications at 5 years included non-proliferative retinopathy in 27%, maculopathy in 25%, proliferative retinopathy in 15%, papillopathy in 26%, cataract in 31%, and glaucoma in 11% of eyes [82]. More recently Echegaray reported their outcomes using episcleral brachytherapy in retinoblastoma, concluding that brachytherapy allowed globe preservation in 82% of eyes overall and was a particularly effective treatment for group B and C eyes. In their series of 11 patients, five developed radiation retinopathy but four of these patients maintained final Snellen VA better than 20/30 [77].

### Vision and amblyopia treatment

Involvement of the fovea by the tumour may suggest a generally poor visual prognosis, however, it is not possible to accurately predict visual acuity. Reports documenting the visual improvement in children with foveal tumours after amblyopia therapy (occlusion) suggests that amblyopia is likely superimposed on organic ocular disease [10, 83]. Thus, reversible amblyopia may coincide with visual loss due to structural damage [52, 84], and teasing one out from the other can at times be challenging.

As described above, it is important to obtain an accurate assessment of visual acuity using preferential looking methods in infants/children and to perform cycloplegic refraction. Glasses need to be given if necessary. If there is evidence of a difference in visual acuity greater than 0.2 logMAR between the eyes, occlusion therapy should be initiated [85]. Watts et al. demonstrated an overall benefit of amblyopia therapy for children with retinoblastoma and no improvement in all children who did not comply [54]. Visual acuity improvement in eyes affected by macular tumours may result from a decrease in the coexisting amblyopia or from time-dependent visual maturation [54]. In support of the former, studies have reported deterioration of vision with cessation of occlusion therapy due to lack of compliance, with no apparent maturational effect [86, 87]. However, as it is unethical to withhold amblyopia therapy, the absence of a control group not exposed to patching in these studies limits our ability to definitively determine the contribution of occlusion versus maturation to visual improvement.

To summarise, occlusion therapy and close follow-up of visual acuity in both eyes can result in improved vision despite anatomically poor prognostic factors (i.e. macular tumour). Parents are often willing to pursue patching when they understand its importance in optimising the potential of their child’s eyes.

### Binocular visual loss

There have been multiple studies assessing monocular visual loss for RB but very few provide prognostic indicators regarding binocular visual loss. Stacey et al reported that 38% of children with bilateral RB have some form of visual impairment. Moreover, 62% avoided visual impairment in a cohort recruited from 2010 to 2014. This is important information to provide parents of children with bilateral disease. Furthermore, there was a correlation between the IIRC group and eventual visual loss with tumour position at the foveola being most relevant [71].

### Severe visual loss and RB

Severe visual loss has been reported in 19% of bilateral retinoblastoma cases. This is a vulnerable group that needs appropriate support as early as possible. Severe visual loss has specific developmental implications and rehabilitation needs that require a concerted effort for this group [88]. It has been shown that the mother–infant interaction is negatively affected by bilateral retinoblastoma associated with severe visual loss.

Binocular visual impairment in children aged 7–10 years has been shown to negatively affect both gross and fine‐motor skills, including balance, upper extremity speed, ball‐catching, and eye‐hand coordination, as well as mobility [71, 89, 90]. Where available, early registration with government agencies devoted to visual impairment rehabilitation can provide patients and families with valuable resources. By assessing the vision as early as possible, visual impairment can be identified and visual rehabilitation will not be delayed [91].

### Nystagmus and RB

An infant with bilateral RB and severe visual loss can present with infantile nystagmus syndrome (INS) from sensory deprivation [92]. This is a useful sign to indicate the severity of disease along with the more commonly known signs (i.e. leukocoria and squint) and usually occurs within the first 6 months of life. Fusion maldevelopment nystagmus syndrome can occur along with INS after 6 months of age [93]. It can be associated with loss of vision in one eye (e.g. enucleation) and good vision in the other eye. In some patients, it can be debilitating with the head turn towards the eye with better vision and reduced vision in that eye due to nystagmus.

### Preferred retinal locus (PRL)

Microperimeters recently emerged to be of great value for quantitative measures of visual field sensitivity alongside a live fundus view, allowing a good understanding of fixation patterns in children with macular diseases [94, 95]. Microperimeters help in understanding the residual visual function in patients with extra-foveal fixation patterns, identifying an eccentric PRL and allowing rehabilitation strategies to take place to improve quality of life [5].

**Fig. 5:**
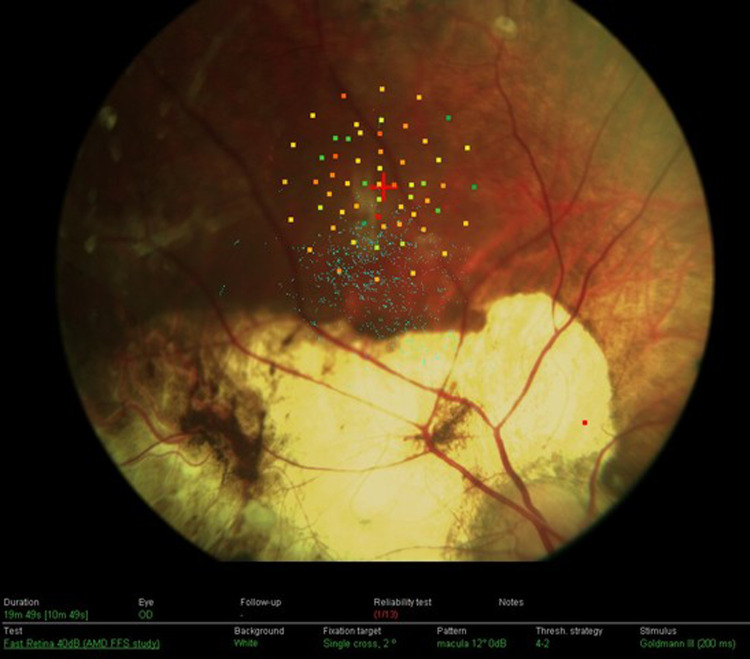
Identification of the preferred retinal locus (PRL) superior to macular tumour scar. Goldmann III stimuli were projected for 200 ms in a 4-2 threshold strategy. The PRL is identified as the centre of the fixation ellipse [94]. (Permission from JAAPOS).

The only child with retinoblastoma who had microperimetry was described by Johnson et al. [96], and the position of the PRL for this patient was superior to the macular tumour (Fig. 5). The PRL had preservation of the anatomical features of the retina and a healthy choroid. The location is similar to other conditions such as Best disease [97]. Applied to children with only one remaining eye, microperimetry combined with SS-OCT testing offers clinicians a new approach in understanding the adaptive mechanisms after retinoblastoma development and may have a role in future visual rehabilitative treatments in older children with the early loss of central vision by retinoblastoma [94, 95].

## Conclusions

With the increased rates of globe salvage, visual outcomes have become an integral part of planning the management for children with retinoblastoma. In some situations, enucleation is still the most appropriate treatment, but with globe-conserving therapy, there is a need for information for patients and physicians alike to make informed decisions. Specialised orthoptic assessments to check visual acuity for children with retinoblastoma has a very important role in the management of those cases. Thus, the prediction of the visual potential for both eyes needs to be tailored for each patient and be part of the parents’ discussion and counselling. Tumour location and grading at presentation are the most important predictors of long-term visual prognosis.

Age-appropriate visual assessments in infants and children with retinoblastoma are important in safety profile data for new treatments. Families can be effectively counselled using the visual outcomes from treatment and where, appropriate visual rehabilitation and support can be provided; in particular liaison with nurseries/schools and local visual impairment teams.
